# Clinical utility of an optoelectronic imaging tracing system for diagnosis of high-grade cervical lesions

**DOI:** 10.3389/fonc.2026.1817331

**Published:** 2026-06-10

**Authors:** Qianjiao Guo, Lixin Liu, Xiaomin Niu, Haifeng Qiu, Hongjun Guo, Wei Feng, Chunfang Wang, Liya Liu, Zheying Liu, Liping Han

**Affiliations:** 1 Department of Gynecology and Obstetrics, The First Affiliated Hospital of Zhengzhou University, Zhengzhou, China; 2 Department of Obstetrics and Gynecology, Peking Union Medical College Hospital, Beijing, China; 3 National Clinical Research Center for Obstetric and Gynecologic Diseases, Beijing, China

**Keywords:** cervical cancer screening, cervical intraepithelial neoplasia, colposcopy, diagnostic accuracy, optoelectronic imaging tracing system (OITS), triage

## Abstract

**Background:**

To evaluate the performance of a novel Optoelectronic Imaging Tracing System (OITS) in diagnosing high-grade cervical intraepithelial neoplasia and to compare its effectiveness with existing methods, including HPV genotyping, liquid-based cytology (LBC), and colposcopy in cervical lesion screening.

**Methods:**

This prospective observational study enrolled 303 women referred for colposcopy between September and December 2024. All participants underwent OITS examination prior to colposcopy, followed by directed cervical biopsy with histopathology as the reference standard. The diagnostic performance of OITS for detecting CIN2+ and CIN3+ lesions was compared with LBC, high-risk HPV (hrHPV) testing, HPV16/18 genotyping, and colposcopic impression. Sensitivity, specificity, predictive values, receiver operating characteristic (ROC) curves, and area under the curve (AUC) were calculated.

**Results:**

Histopathology identified 82 cases of CIN2+ and 34 cases of CIN3 +. OITS demonstrated the highest diagnostic accuracy for CIN2+ detection, with an AUC of 0.798 (95% CI: 0.762–0.834), sensitivity of 97.6% (95% CI: 94.2-100), specificity of 62.0% (95% CI: 55.6-68.4), and a negative predictive value of 98.6% (95% CI: 96.6-100.5). DeLong’s test for correlated ROC curves confirmed that the diagnostic accuracy of OITS was statistically significantly superior to other methods evaluated (all P<0.05). OITS positivity increased with lesion severity and reached 100% in CIN3 and invasive cancer. Across stratified subgroups defined by HPV status, cytology, and colposcopic impression, OITS consistently maintained sensitivity above 93%, outperforming all comparator methods.

**Conclusions:**

OITS shows excellent and stable performance in identifying high-grade cervical lesions among women referred for colposcopy. Its high sensitivity, strong rule-out capability, and objective assessment suggest that OITS may serve as an effective adjunct or alternative triage tool to optimize cervical cancer screening and referral strategies.

## Introduction

Cervical cancer remains a major global health issue, primarily caused by persistent infection with high-risk human papillomavirus (hrHPV) (1, 2). Epidemiological studies indicate that approximately 80% of sexually active women will contract HPV at some point in their lives, with about 10% developing persistent infections that may lead to cervical precancerous lesions or cancer (3). Globally, cervical cancer is the fourth most common cancer among women, with 604,000 new cases and 342,000 deaths reported in 2020, highlighting its substantial disease burden (4). Notably, cervical cancer is currently the only human malignancy that is both preventable and curable when detected and treated at a precancerous stage (5). The World Health Organization has launched a global strategy to eliminate cervical cancer, which includes HPV vaccination, screening women with high-performance tests, early diagnosis, and timely treatment of high-grade cervical intraepithelial neoplasia and cancer (6). The success of this strategy relies heavily on accurate identification of women at true risk for disease progression and on a profound understanding of the dynamic biological process underlying cervical carcinogenesis (7).

Current domestic and international screening and diagnostic methods for cervical lesions follow the classic three-step model (cervical cytology and HPV testing, colposcopy, and cervical biopsy pathology), which plays a significant role in detecting early lesions and initiating timely treatment (8, 9). However, this multistep approach is inherently complex and time-consuming, increasing the risk of loss to follow-up, particularly in economically underdeveloped regions. In high-income countries, cytology triage for HPV-positive women before confirming cervical cytology abnormalities is an effective primary approach (10, 11). Nevertheless, the successful implementation of this strategy requires well-organized healthcare infrastructure and highly trained professionals, including pathologists, cytopathologists, laboratory scientists, and experienced colposcopists, limiting its scalability and consistency (12). Furthermore, conventional colposcopic assessment remains subjective and operator-dependent, with limited ability to reliably distinguish clinically significant lesions, particularly in women with equivocal screening results (13).Therefore, there is a clinical need for an adjunct technology capable of real-time, objective, and high-specificity differentiation between high-grade and low-grade squamous intraepithelial lesions or normal tissue to improve triage efficiency and enable rapid, accurate diagnosis (14).

Optoelectronic Imaging Tracing System (OITS) represents an emerging technology that combines fluorescence-based photoelectric detection with real-time imaging and automated analysis. OITS integrates optical and electrical detection, using four light wavelengths (ultraviolet, green, red, and infrared) to measure tissue optical properties and electrical parameters. And distinguishes between different kinds of cervical epithelium through advanced algorithms, allowing the determination of the degree of pathology and the physiological status of the tissue (15, 16). The high sensitivity of the OITS allows rapid tissue classification and determination of diagnostic results through fiber optic electrodes and computer analysis. It features simplicity, low cost, and non-invasiveness, providing clinicians with pathophysiological information beyond visual observation, demonstrating significant diagnostic potential. Preliminary studies have suggested that as a new-generation optoelectronic detection system, OITS has been demonstrated to serve as an effective triage tool for HPV-positive women (17).

In this study, we aimed to assess the diagnostic accuracy of OITS for the detection of CIN2+ and CIN3+ lesions in a colposcopy-referred population, comparing its performance with LBC, hrHPV genotyping, and conventional colposcopy. We further explored the clinical utility of OITS in stratified subgroups, including different HPV genotypes, cytology results, and colposcopic impressions, to evaluate its potential role as an adjunct triage tool within existing cervical cancer screening pathways.

## Methods

### Study subjects

This was a prospective observational study conducted between September 2024 and December 2024 at the Department of Gynecology, the First Affiliated Hospital of Zhengzhou University. A total of 303 women who were referred for colposcopic examination after completing liquid-based cytology (LBC) and human papillomavirus (HPV) testing were enrolled. All included participants underwent evaluation with the OITS prior to colposcopic examination. During colposcopic examination, cervical biopsy specimens were obtained from all sites suspected of abnormality, and histopathological diagnosis was used as the reference standard. This study was approved by the Ethics Committee of Peking Union Medical College Hospital, Chinese Academy of Medical Sciences (approval number: K6534). Written informed consent was obtained from all participants prior to enrollment, and the study was conducted in accordance with the Declaration of Helsinki. The study was registered at ClinicalTrials.gov (registration number: NCT06491888; registration date: July 1, 2024).

### Inclusion and exclusion criteria

Women were eligible for enrollment if they met all of the following criteria: Age and sexual activity: Aged ≥18 and ≤65 years with a history of sexual activity. Anatomical eligibility: Intact cervix with no prior surgical removal or structural abnormality precluding examination. Screening indication: Had documented cervical cancer screening results meeting at least one of the following triage criteria: HPV16/18 genotype positive, regardless of cytology result; or other high-risk HPV genotype positive (non-16/18) with concurrent cytology result of atypical squamous cells of undetermined significance (ASCUS) or worse (≥ASCUS), as determined by liquid-based cytology (LBC). Colposcopy referral: Referred for colposcopic evaluation based on the above screening results in accordance with current national clinical guidelines. Informed consent: Willing and able to provide written informed consent after receiving a full explanation of study procedures, risks, and benefits. Absence of prior cervical malignancy: No personal history of cervical cancer or prior treatment for invasive cervical disease.

Exclusion criteria: Pregnancy or less than 4 months postpartum; Cervical malformation or lower genital tract infection; Menstruation or coagulation disorders; History of hysterectomy or cervical surgery; Received experimental photodynamic therapy or otherwise exposed to photosensitizing drugs, or have photosensitive diseases (e.g., porphyria, lupus erythematosus, etc.); Concurrent cancer at other sites; Presence of definite immunosuppressive disease or use of immunosuppressive drugs; Patients deemed unsuitable for the study by the investigators due to inability to follow up, etc.

### HPV testing and cytology testing

Cytologists classified liquid-based cytology test results using the 2014 Bethesda system (18). In this study, cytological diagnoses were categorized as negative for intraepithelial lesion or malignancy (NILM), atypical squamous cells of undetermined significance (ASCUS), low-grade squamous intraepithelial lesions (LSIL), atypical squamous cells—cannot exclude HSIL (ASC-H), high-grade squamous intraepithelial lesions (HSIL), or atypical glandular cells (AGC). For analytical purposes, cytology results were dichotomized as NILM versus ≥ASCUS.

HPV testing was performed using an *in vitro* polymerase chain reaction-based quantitative method for detecting hrHPV DNA, capable of detecting hrHPV types including HPV16, HPV18, and non-16/18 hrHPV (31, 33, 35, 39, 45, 51, 52, 56, 58, 59, 66, and 68). HPV results were categorized as HPV16/18 positive, non-16/18 hrHPV positive, HPV (-), or unknown. In this study, all enrolled women were either hrHPV-infected or HPV (-) but with LBC was ASCUS or worse.

### Optoelectronic imaging tracing system examination

The fluorescence-based photoelectric cervical lesion detector (OITS) produced by Su Zhou Surgi-Master High Tech Co., Ltd. (Suzhou, China) was used. OITS integrates optical and electrical detection using four light wavelengths: ultraviolet, green, red, and infrared to simultaneously measure tissue fluorescence, reflectance, and electrical impedance properties of cervical epithelium. The hardware system comprises a 4.5 mm fiber-optic probe with a sterilized contact sensor, an integrated ultra-high-definition vaginal camera, a built-in colposcope for real-time visual guidance, and a foot pedal for operator-controlled measurement triggering. The one- time contact fiber-optic electrode has an elbow design, which can closely fit the arc of the cervical surface and improve the efficiency of diffuse reflection and fluorescence return. The software component incorporates a proprietary automated classification algorithm that analyzes multi-spectral signals acquired from each measurement point and classifies cervical tissue as normal or abnormal in real time. The typical duration of the OITS examination is approximately 3–5 minutes per patient under routine conditions.

Operators performed cervical examinations according to the instrument’s instructions. At each research center, primary investigators received at least 4 hours of training on using the OITS and performed at least 5 colposcopic procedures with the system. With the subject in the lithotomy position and the cervix fully exposed, secretions on the cervical surface were wiped clean with a dry cotton ball. A 4.5mm fiber-optic electrode equipped with a sterilized sensor was used to sequentially measure various points on the cervical surface under white light and an attached ultra-high-definition vaginal camera, and images were recorded. Thirty-six sites on the cervical surface (exploring 3 circles along the 12 o’clock direction) were routinely measured for detection. Using a foot pedal to trigger measurements, precise mapping of the detected points was achieved via visual enhancement technology under the assistance of the built-in colposcope through real-time imaging. Finally, the software algorithm automatically judged and marked whether the detected points had lesions.

### Traditional colposcopy

Following the OITS examination, traditional colposcopy based on acetic acid staining and Lugol’s iodine reagent was performed to assess suspicious lesion sites and obtain biopsy samples, with endocervical curettage performed if necessary. After the procedure, original data including test data and images were copied for retention, and pathology slide preparation and reporting results were tracked.

### Histopathological examination

All biopsy specimens, conducted by the same group of pathologists and reviewed by an experienced senior pathologist. Pathological diagnoses included: Benign changes; CIN1; CIN2; CIN3; Adenocarcinoma *in situ* (AIS); and Invasive cancer.

### Statistical analysis

Statistical analyses were performed using SPSS version 26.0 (IBM Corp., Armonk, NY, USA) and R software (version 4.1.2). Count data are expressed as number (n) and percentage (%). Continuous variables were tested for normality using the Kolmogorov–Smirnov test and are presented as median and interquartile range (IQR). Categorical variables are expressed as counts and percentages. Diagnostic performance metrics, including sensitivity, specificity, positive predictive value (PPV), and negative predictive value (NPV), were calculated with 95% confidence intervals (CIs). Receiver operating characteristic (ROC) curves were constructed, and the area under the curve (AUC) was used to assess discriminative ability. Comparisons between correlated ROC curves were performed using DeLong’s test. Differences in paired proportions were assessed using McNemar’s test where appropriate. All statistical tests were two-sided, and a P value <0.05 was considered statistically significant.

## Results

**Figure 1:**
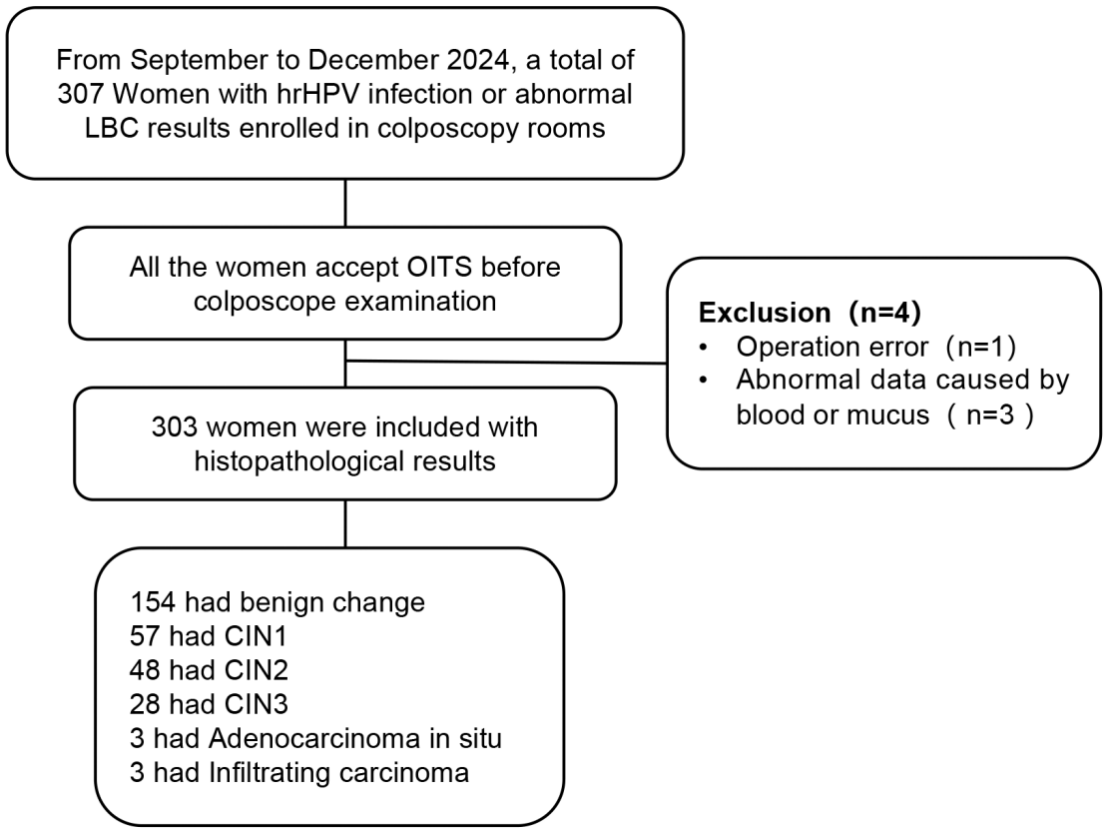
Flowchart of study procedures. CIN, Cervical intraepithelial neoplasia; hrHPV, high risk human papillomavirus; LCB, Liquid-based cytology test; OITS, Optoelectronic Imaging Tracing System.

A total of 303 women referred for colposcopic examination were consecutively enrolled in this study, and the participant flow is illustrated in **Figure 1**. The median age of the overall cohort was 42 years (IQR; 35–51). HPV genotyping revealed that 106 women (35.0%) were infected with HPV16/18, 184 (60.7%) with non-HPV16/18 high-risk types, 12 (4.0%) were HPV (-), and 1 (0.3%) had an unknown HPV status. Regarding cytology, 73 women (24.1%) had negative for NILM, 119 (39.3%) had ASCUS, 69 (22.8%) had LSIL, 17 (5.6%) had ASC-H, 13 (4.3%) had HSIL, and 4 (1.3%) had AGC. Most participants were premenopausal (227/303, 74.9%), while 76 (25.1%) were postmenopausal. Transformation zone (TZ) types I, II, and III accounted for 20.5%, 18.8%, and 60.4% of cases, respectively, with a statistically significant distribution difference between OITS(+) and OITS (-) groups (p = 0.009). On colposcopic impression, 81 women (26.7%) exhibited normal findings, 179 (59.1%) had LSIL, 39 (12.9%) had HSIL, and 4 (1.3%) had infiltrating carcinoma, with OITS (+) women showing a significantly higher proportion of high-grade colposcopic impressions (p < 0.001)Histopathological examination demonstrated benign or inflammatory changes in 154 women (50.8%), CIN1 in 67 (22.1%), CIN2 in 48 (15.8%), CIN3 in 28 (9.2%), AIS in 3 (1.0%), and invasive squamous cell carcinoma in 3 (1.0%). High-grade cervical lesions (CIN2+) were significantly more prevalent in the OITS(+) group compared with the OITS (-) group (p < 0.001) (**Table 1**).

**Table 1:** Clinical characteristics of the participants.

Characteristics	Level	Overall	OITS-	OITS+	p
	n	303	139	164	
AGE (median [IQR])		42 [35, 51]	44 [36, 53]	38 [3, 50]	0.034
HPV types (%)	HPV-	12 (4.0)	7 (5.0)	5 (3.0)	0.636
	HPV1618	106 (35.0)	47 (33.8)	59 (36.0)	
	nonHPV1618	184 (60.7)	85 (61.2)	99 (60.4)	
	Unknown	1 (0.3)	0 (0.0)	1 (0.6)	
TCT results (%)	NILM	73 (24.1)	39 (28.1)	34 (20.7)	0.005
	ASCUS	119 (39.3)	58 (41.7)	61 (37.2)	
	LSIL	69 (22.8)	28 (20.1)	41 (25.0)	
	ASC-H	17 (5.6)	5 (3.6)	12 (7.3)	
	HSIL	13 (4.3)	1 (0.7)	12 (7.3)	
	AGC	4 (1.3)	1 (0.7)	3 (1.8)	
	Unknown	8 (2.6)	7 (5.0)	1 (0.6)	
Menopause (%)	Premenopausal	227 (74.9)	99 (71.2)	128 (78.0)	0.218
	Postmenopause	76 (25.1)	40 (28.8)	36 (22.0)	
TZ (%)	I	62 (20.5)	20 (14.4)	42 (25.6)	0.009
	II	57 (18.8)	21 (15.1)	36 (22.0)	
	III	183 (60.4)	98 (70.5)	85 (51.8)	
	Unknown	1 (0.3)	0 (0.0)	1 (0.6)	
Colposcopy image (%)	Normal	81 (26.7)	53 (38.1)	28 (17.1)	<0.001
	LSIL	179 (59.1)	78 (56.1)	101 (61.6)	
	HSIL	39 (12.9)	8 (5.8)	31 (18.9)	
	Infiltrating carcinoma	4 (1.3)	0 (0.0)	4 (2.4)	
Pathology results (%)	Benign change	154 (50.8)	101 (72.7)	53 (32.3)	<0.001
	CIN1	67 (22.1)	36 (25.9)	31 (18.9)	
	CIN2	48 (15.8)	2 (1.4)	46 (28.0)	
	CIN3	28 (9.2)	0 (0.0)	28 (17.1)	
	AIS	3 (1.0)	0 (0.0)	3 (1.8)	
	Infiltrating carcinoma (squamous cell carcinoma)	3 (1.0)	0 (0.0)	3 (1.8)	

AIS, Adenocarcinoma *in situ*; ASCUS, Atypical squamous cells of undetermined significance; CIN, Cervical intraepithelial neoplasia; hrHPV, High-risk human papillomavirus; IQR, Interquartile range; LBC, Liquid-based cytology test; NILM, Negative for intraepithelial lesion or malignancy; OITS, Optoelectronic Imaging Tracing System; ≥LSIL, Low-grade squamous intraepithelial lesion or worse; TZ, Transformation zone.

**Figure 2:**
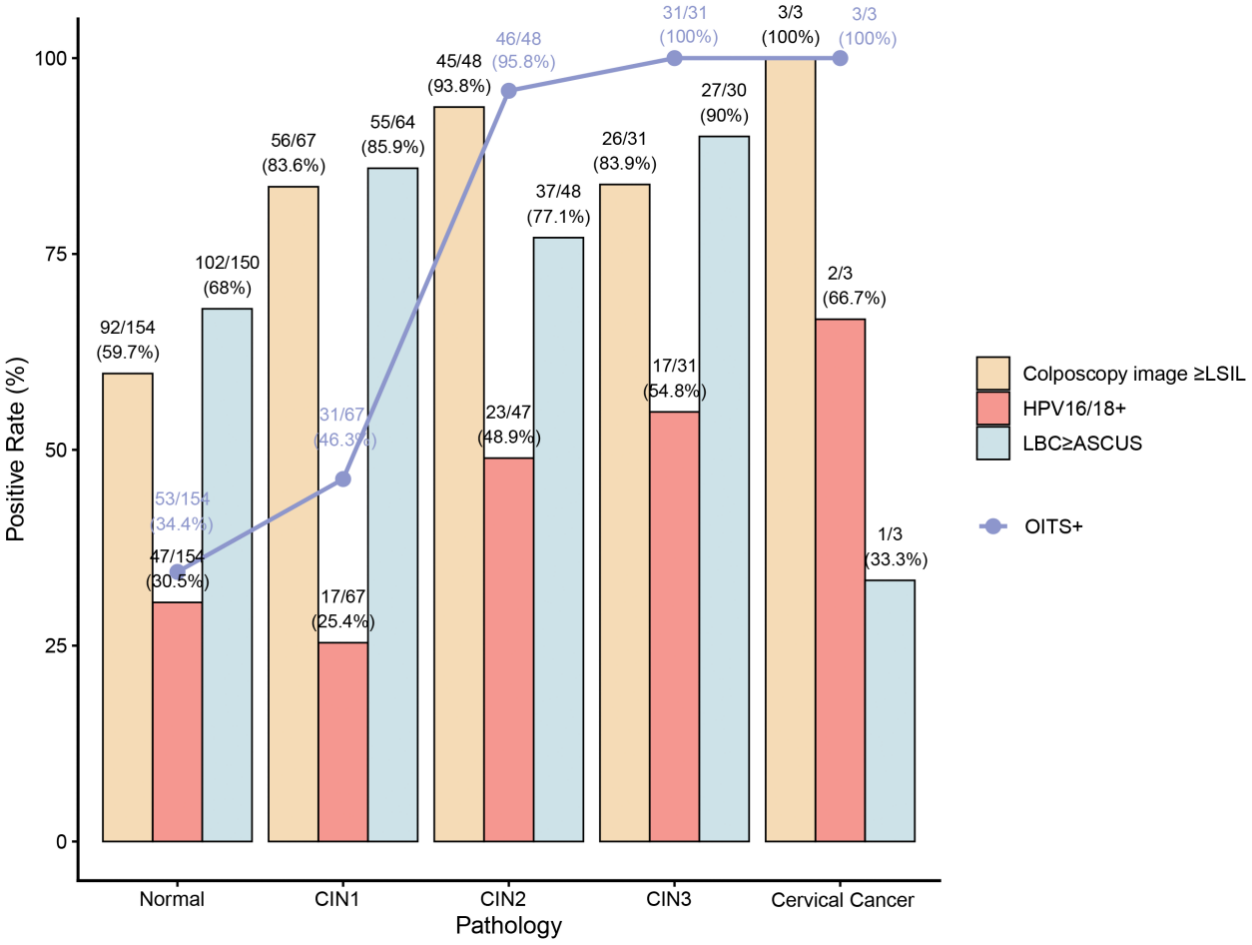
Positive rates of different screening methods for different histological types. CIN, Cervical intraepithelial neoplasia; Colposcopy image ≥ LSIL, Colposcopy image test results suggesting low-grade squamous intraepithelial lesion or worse; HPV 16/18(+), Human papillomavirus 16 or 18 types positive; LCB≥ASCUS, Liquid-based cytology test results suggesting atypical squamous cells of undetermined significance or worse; OITS, Optoelectronic Imaging Tracing System.

To compare the diagnostic performance of HPV testing, LBC testing, colposcopy image assessment, and the OITS assay across the full spectrum of cervical histopathology, the positivity rates of each method stratified by pathological severity are summarized in **Figure 2**. Notably, OITS (+) increased monotonically with lesion severity, reaching 100% in both CIN3 and invasive cervical cancer, highlighting its strong capability for identifying high-grade and malignant lesions.

**Figure 3:**
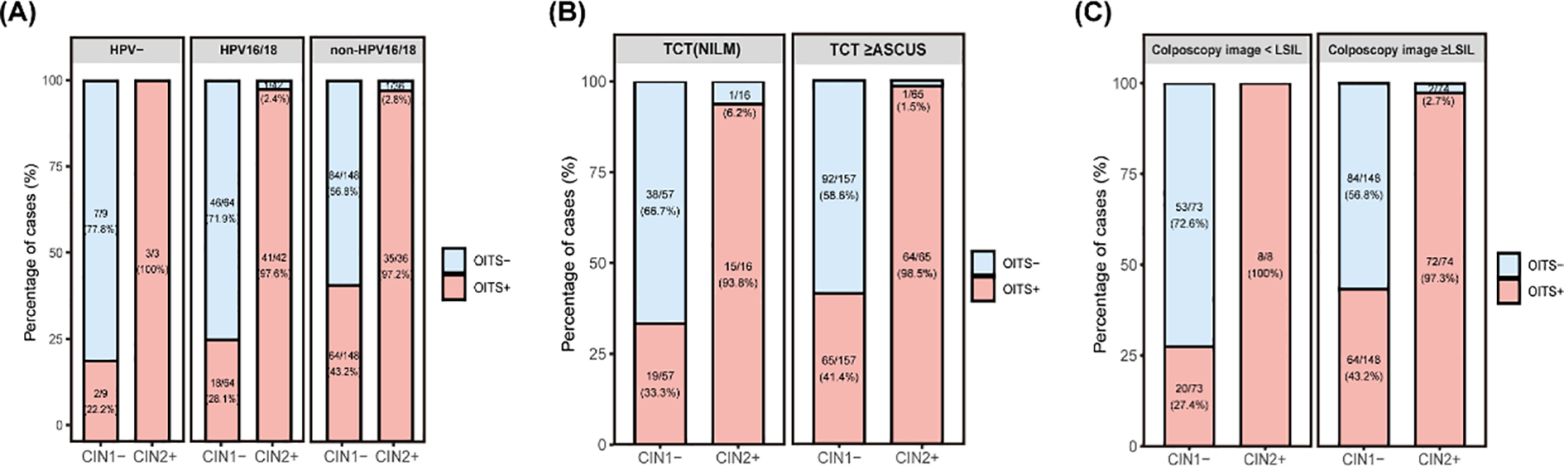
OITS performance in CIN2+ detection by clinical subgroups. CIN, Cervical intraepithelial neoplasia; CIN1-, include pathology result was naomal and CIN1; CIN2+, Cervical intraepithelial neoplasia grade 2 or worse; Colposcopy image ≥ LSIL, Colposcopy image test results suggesting low-grade squamous intraepithelial lesion or worse; HPV 16/18(+), Human papillomavirus 16 or 18 types positive; LCB≥ASCUS, Liquid-based cytology test results suggesting atypical squamous cells of undetermined significance or worse; non-16/18 hrHPV(+), Infection with one or more strains of HPV 31, 33, 35, 39, 45, 51, 52, 56, 58, 59, 66, or 68; OITS, Optoelectronic Imaging Tracing System.

To further evaluate the clinical utility of the OITS test, we stratified the study population according to HPV status, LBC results, and colposcopy image findings, and analyzed the association between OITS (+) and histologically confirmed CIN2+ lesions (**Figure 3**). Across all stratified subgroups, the OITS assay demonstrated consistently high sensitivity for the detection of CIN2+, with positivity rates ranging from 93.8% to 100% among women with confirmed disease. Specifically, among HPV (-) women, all CIN2+ cases (100%, 3/3) were OITS (+), whereas only 22.2% (2/9) of CIN1- (women with pathological results were benign or CIN1) cases tested positive. In women positive for HPV16/18, OITS detected 97.6% (41/42) of CIN2+ lesions, compared with 28.1% (18/64) positivity among CIN1- cases. Similarly, among women infected with non-16/18 high-risk HPV types, 97.2% (35/36) of CIN2+ cases were OITS (+), while 43.2% (64/148) of CIN1- cases showed positive results. Stratification by cytology yielded comparable results. Among women with NILM, OITS was positive in 93.8% (15/16) of CIN2+ cases and in 33.3% (19/57) of CIN1- cases. In those with abnormal cytology (≥ASCUS), OITS identified 98.5% (64/65) of CIN2+ lesions, whereas 41.4% (65/157) of CIN1- cases were OITS (+). When stratified by colposcopy image assessment, all CIN2+ cases (100%, 8/8) with images suggestive of <LSIL were OITS (+), compared with 27.4% (20/73) of CIN1- cases. Among women with colposcopic impressions of ≥LSIL, OITS (+) reached 97.3% (72/74) in CIN2+ cases and 43.2% (64/148) in CIN1- cases. Collectively, these findings demonstrate that OITS maintains robust sensitivity for CIN2+ detection across diverse clinical scenarios, including low-risk HPV status, negative cytology, and low-grade colposcopic impressions. Collectively, these findings demonstrate that OITS maintains robust sensitivity for CIN2+ detection across diverse clinical scenarios, including low-risk HPV status, negative cytology, and low-grade colposcopic impressions.

**Figure 4:**
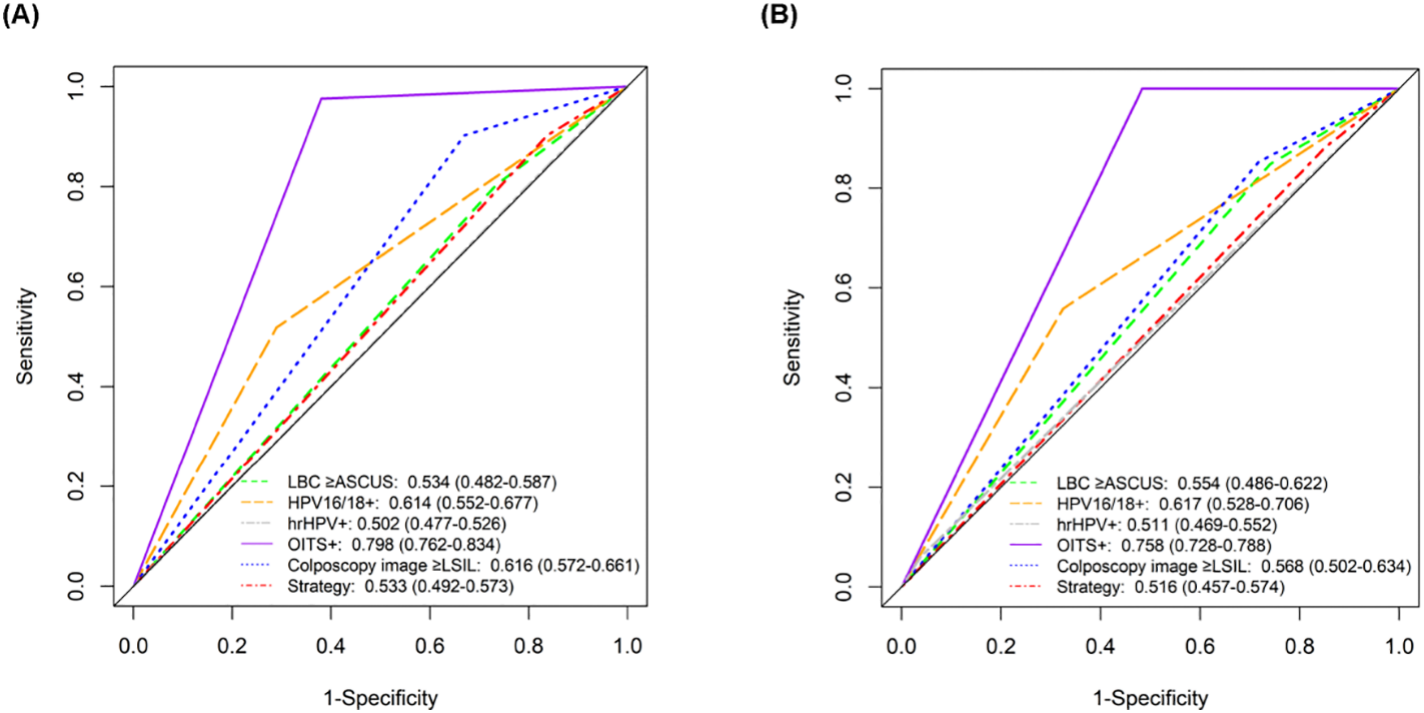
ROC curves for different screening methods in detecting CIN2+ **(A)** and CIN3+ **(B)**. DeLong’s test was used to compare the ROC curves of OITS with those of other diagnostic methods. CIN: Cervical intraepithelial neoplasia; Colposcopy image ≥ LSIL, Colposcopy image test results suggesting low-grade squamous intraepithelial lesion or worse; HPV 16/18(+), Human papillomavirus 16 or 18 types positive; LCB≥ASCUS, Liquid-based cytology test results suggesting atypical squamous cells of undetermined significance or worse; non-16/18 hrHPV(+), Infection with one or more strains of HPV 31, 33, 35, 39, 45, 51, 52, 56, 58, 59, 66, or 68; OITS, Optoelectronic Imaging Tracing System; Strategy, Women who test positive for HPV16/18, as well as those positive for non-16/18 high-risk HPV and with liquid-based cytology test results suggesting atypical squamous cells of undetermined significance or worse, should be referred for colposcopy.

The diagnostic performance of different screening methods for detecting CIN2+ and CIN3+ is summarized in **Tables 2**, **3**, and illustrated by ROC curves (**Figure 4**). Among all evaluated methods, the OITS demonstrated the highest overall performance. For CIN2+ versus Normal/CIN1, the AUC of OITS was 0.798 (95% CI: 0.762–0.834), with a sensitivity of 97.6% (95% CI: 94.2–100) and specificity of 62.0% (95% CI: 55.6–68.4). The PPV and NPV were 48.8% (95% CI: 41.1–56.4) and 98.6% (95% CI: 96.6–100.5), respectively, indicating excellent ability to detect CIN2+ lesions while minimizing false negatives. By comparison, LBC with results ≥ASCUS achieved an AUC of 0.534 (95% CI: 0.482–0.587), with high sensitivity (80.2%; 95% CI: 71.6–88.9) but low specificity (26.6%; 95% CI: 20.7–32.6). HPV16/18 positivity showed moderate discriminative ability (AUC = 0.614, 95% CI: 0.552–0.677) with lower sensitivity (51.9%; 95% CI: 41–62.7) and higher specificity (71.0%; 95% CI: 65.1–77). High-risk HPV testing had very high sensitivity (96.3%; 95% CI: 92.2–100) but extremely low specificity (4.1%; 95% CI: 1.5–6.7), limiting its practical utility. Colposcopy images suggestive of ≥LSIL achieved an AUC of 0.616 (95% CI: 0.572–0.661), with sensitivity of 90.2% (95% CI: 83.8–96.7) and specificity of 33.0% (95% CI: 26.8–39.2). A combined triage strategy+ (Women who test positive for HPV16/18, as well as those positive for non-16/18 high-risk HPV with abnormal cytology) yielded an AUC of 0.533 (95% CI: 0.492–0.573), with 90.2% sensitivity and 16.3% specificity. DeLong’s test for correlated ROC curves confirmed that the diagnostic accuracy of OITS was statistically significantly superior to all other methods evaluated (Z = 8.14, Z = 4.86, Z = 6.41, Z = 9.71, and Z = 13.36, all P<0.05).

**Table 2:** Diagnostic performance of different detection methods for CIN2 +.

Characteristics	AUC (95% CI)	SEN (95% CI)	SPE (95% CI)	PPV (95% CI)	NPV (95% CI)
CIN2+ *vs*. Normal/CIN1					
LBC ≥ ASCUS	0.534 (0.482-0.587)	80.2 (71.6-88.9)	26.6 (20.7-32.6)	29.3 (23.3-35.3)	78.1 (68.6-87.6)
HPV16/18 (+)	0.614 (0.552-0.677)	51.9 (41-62.7)	71 (65.1-77)	39.6 (30.3-48.9)	80.1 (74.5-85.7)
hrHPV (+)	0.502 (0.477-0.526)	96.3 (92.2-100)	4.1 (1.5-6.7)	26.9 (21.8-32)	75 (50.5-99.5)
OITS (+)	0.798 (0.762-0.834)	97.6 (94.2-100)	62 (55.6-68.4)	48.8 (41.1-56.4)	98.6 (96.6-100.5)
Colposcopy image ≥ LSIL	0.616 (0.572-0.661)	90.2 (83.8-96.7)	33 (26.8-39.2)	33.3 (27.1-39.5)	90.1 (83.6-96.6)
Strategy (+)	0.533 (0.492-0.573)	90.2 (83.8-96.7)	16.3 (11.4-21.2)	28.6 (23.1-34.1)	81.8 (70.4-93.2)
CIN2+ *vs*. Normal					
LBC ≥ ASCUS	0.561 (0.504-0.619)	80.2 (71.6-88.9)	32 (24.5-39.5)	38.9 (31.5-46.3)	75 (64.4-85.6)
HPV16/18 (+)	0.607 (0.541-0.672)	51.9 (41-62.7)	69.5 (62.2-76.8)	47.2 (36.8-57.6)	73.3 (66.1-80.5)
hrHPV (+)	0.504 (0.478-0.531)	96.3 (92.2-100)	4.5 (1.3-7.8)	34.7 (28.4-40.9)	70 (41.6-98.4)
OITS (+)	0.816 (0.775-0.857)	97.6 (94.2-100)	65.6 (58.1-73.1)	60.2 (51.8-68.5)	98.1 (95.4-100.7)
Colposcopy image ≥ LSIL	0.653 (0.602-0.703)	90.2 (83.8-96.7)	40.3 (32.5-48)	44.6 (37-52.1)	88.6 (81.1-96)
Strategy (+)	0.545 (0.501-0.59)	90.2 (83.8-96.7)	18.8 (12.7-25)	37.2 (30.5-43.9)	78.4 (65.1-91.6)

AUC: Area Under Curve; CIN2+: Cervical intraepithelial neoplasia grade 2 or worse; Colposcopy image ≥ LSIL: Colposcopy image test results suggesting low-grade squamous intraepithelial lesion or worse; HPV 16/18(+): Human papillomavirus 16 or 18 types positive; hrHPV(+): High-risk human papillomavirus; LCB≥ASCUS: Liquid-based cytology test results suggesting atypical squamous cells of undetermined significance or worse; NPV: Negative predictive value; OITS: Optoelectronic Imaging Tracing System; PPV: Positive predictive value; Strategy: Women who test positive for HPV16/18, as well as those positive for non-16/18 high-risk HPV and with liquid-based cytology test results suggesting atypical squamous cells of undetermined significance or worse, should be referred for colposcopy.

**Table 3:** Diagnostic performance of different detection methods for CIN3 +.

Characteristics	AUC (95% CI)	SEN (95% CI)	SPE (95% CI)	PPV (95% CI)	NPV (95% CI)
CIN3+ *vs*. Normal/CIN1/CIN2					
LBC ≥ ASCUS	0.554 (0.486-0.622)	84.8 (72.6-97.1)	26 (20.6-31.3)	12.6 (8.2-17)	93.2 (87.4-98.9)
HPV16/18 (+)	0.617 (0.528-0.706)	55.9 (39.2-72.6)	67.5 (61.9-73.1)	17.9 (10.6-25.2)	92.3 (88.6-96.1)
hrHPV (+)	0.489 (0.448-0.531)	94.1 (80.9-98.4)	3.7 (2.0-6.7)	11.0 (7.9-15.1)	88.7 (85.2-92.3)
OITS (+)	0.758 (0.728-0.788)	100 (100-100)	51.7 (45.7-57.6)	20.7 (14.5-26.9)	100 (100-100)
Colposcopy image ≥ LSIL	0.568 (0.502-0.634)	85.3 (73.4-97.2)	28.3 (22.9-33.6)	13.1 (8.6-17.5)	93.8 (88.6-99.1)
Strategy (+)	0.516 (0.457-0.574)	88.2 (77.4-99.1)	14.9 (10.6-19.1)	11.6 (7.7-15.5)	90.9 (82.4-99.4)
CIN3+ *vs*. Normal/CIN1					
LBC ≥ ASCUS	0.557 (0.489-0.626)	84.8 (72.6-97.1)	26.6 (20.7-32.6)	15.1 (10-20.3)	91.9 (85.2-98.7)
HPV16/18 (+)	0.635 (0.545-0.724)	55.9 (39.2-72.6)	71 (65.1-77)	22.9 (13.9-31.9)	91.3 (87.1-95.5)
hrHPV (+)	0.491 (0.449-0.533)	94.1 (80.9-98.4)	4.07 (2.16-7.56)	13.1 (9.45-17.9)	81.8 (52.3-94.8)
OITS (+)	0.81 (0.778-0.842)	100 (100-100)	62 (55.6-68.4)	28.8 (20.6-37)	100 (100-100)
Colposcopy image ≥ LSIL	0.592 (0.524-0.66)	85.3 (73.4-97.2)	33 (26.8-39.2)	16.4 (10.9-21.8)	93.6 (88.2-99)
Strategy (+)	0.523 (0.462-0.583)	88.2 (77.4-99.1)	16.3 (11.4-21.2)	14 (9.3-18.6)	90 (80.7-99.3)
CIN3+ *vs*. Normal					
LBC ≥ ASCUS	0.584 (0.512-0.657)	84.8 (72.6-97.1)	32 (24.5-39.5)	21.5 (14.5-28.6)	90.6 (82.7-98.4)
HPV16/18 (+)	0.627 (0.535-0.719)	55.9 (39.2-72.6)	69.5 (62.2-76.8)	28.8 (17.9-39.7)	87.7 (81.9-93.5)
hrHPV (+)	0.493 (0.45-0.537)	94.1 (80.9-98.4)	4.5 (2.22-9.08)	17.9 (12.9-24.1)	77.8(45.2-93.7)
OITS (+)	0.828 (0.79-0.866)	100 (100-100)	65.6 (58.1-73.1)	39.1 (28.8-49.3)	100 (100-100)
Colposcopy image ≥ LSIL	0.628 (0.556-0.7)	85.3 (73.4-97.2)	40.3 (32.5-48)	24 (16.4-31.6)	92.5 (86.2-98.8)
Strategy (+)	0.535 (0.472-0.598)	88.2 (77.4-99.1)	18.8 (12.7-25)	19.4 (13.1-25.6)	87.9 (76.7-99)

AUC: Area Under Curve; CIN2+: Cervical intraepithelial neoplasia grade 2 or worse; Colposcopy image ≥ LSIL, Colposcopy image test results suggesting low-grade squamous intraepithelial lesion or worse; HPV 16/18(+), Human papillomavirus 16 or 18 types positive; hrHPV(+), High-risk human papillomavirus; LCB≥ASCUS, Liquid-based cytology test results suggesting atypical squamous cells of undetermined significance or worse; NPV, Negative predictive value; OITS, Optoelectronic Imaging Tracing System; PPV, Positive predictive value; Strategy, Women who test positive for HPV16/18, as well as those positive for non-16/18 high-risk HPV and with liquid-based cytology test results suggesting atypical squamous cells of undetermined significance or worse, should be referred for colposcopy.

When considering CIN2+ versus Normal only, OITS maintained the highest discriminative power (AUC = 0.816; 95% CI: 0.775–0.857), with 97.6% sensitivity and 65.6% specificity, as well as a PPV of 60.2% and NPV of 98.1%, outperforming other methods. LBC ≥ASCUS, HPV16/18 positivity, hrHPV positivity, colposcopy ≥LSIL, and Strategy+ showed lower AUCs (0.561, 0.607, 0.504, 0.653, and 0.545, respectively) and variable sensitivity and specificity, consistent with findings for CIN2+ versus Normal/CIN1. The results for the detection of CIN3+ using the different methods are presented in **Table 3**, which shows findings similar to those reported for CIN2 +. Briefly, Optoelectronic Imaging Tracing System emerged as the most effective method for identifying CIN3 +.

## Discussion

In this study, we comprehensively evaluated the clinical performance of the OITS in detecting high-grade cervical lesions among women referred for colposcopy and compared its diagnostic accuracy with that of conventional screening and triage approaches, including HPV testing, liquid-based cytology, and colposcopy image assessment. Our findings demonstrate that OITS consistently outperformed all comparator methods in identifying CIN2+ and CIN3+ lesions, achieving the highest AUC values, excellent sensitivity, and a particularly high negative predictive value across diverse clinical subgroups.

A key finding of this study is the remarkably robust and stable sensitivity of OITS for CIN2+ detection. Sensitivity exceeded 97% in the overall cohort and remained above 93% across all stratified analyses, including HPV (-) women, those with NILM cytology, and women with colposcopic impressions suggestive of less than LSIL. Furthermore, OITS (+) increased monotonically with lesion severity, reaching 100% in cases of CIN3 and invasive cervical cancer, indicating a strong correlation between optoelectronic signal alterations and histopathological progression. This finding establishes a foundation for the role of OITS in cervical cancer screening, whose primary objective of cervical cancer screening is the early detection of high-grade precancerous lesions (CIN2, CIN3, and AIS) that carry a substantial risk of progression to invasive cancer (19–21).

Currently, TCT and hrHPV testing constitute the cornerstone of cervical cancer screening (22, 23). However, the single-round sensitivity of TCT for CIN2+ lesions ranges from approximately 50% to 80%, while hrHPV testing, although highly sensitive, suffers from relatively low specificity (24–26). Although hrHPV testing and cytology demonstrated acceptable sensitivity in this study (80.2% and 96.3% for CIN2+, 84.8% and 94.1% for CIN3+), their limited specificity resulted in suboptimal discriminatory performance (26.6% and 4.1% for CIN2+, 26.0% and 3.7% for CIN3+). This limitation is largely attributable to the transient nature of most HPV infections, which are frequently cleared by the host immune system without causing clinically significant cervical lesions (27). Moreover, hrHPV testing reflects viral infection status rather than cellular transformation, necessitating additional triage tests such as cytology or HPV genotyping, thereby increasing costs and clinical burden (28). In contrast, OITS enables rapid, multi-parameter assessment of cervical epithelial cells, offering advantages in operational simplicity, speed, and diagnostic accuracy. Our results showed that the AUC of OITS for detecting CIN2+ was 0.798 (95% CI: 0.762–0.834), which was significantly higher than that of HPV testing (0.502, 95% CI: 0.477–0.526) and cytology (0.534, 95% CI: 0.482–0.587). This indicates that OITS provides a more accurate and balanced discrimination between high-grade and non–high-grade cervical lesions. Consequently, OITS may partially substitute for TCT and serve as a novel triage tool within the colposcopy referral pathway.

Notably, even among women infected with non-16/18 hrHPV types, a subgroup widely recognized as a clinical management challenge, OITS maintained a sensitivity exceeding 97% for CIN2+ detection. This performance compares favorably with previously reported molecular or image-based adjunctive tests, many of which exhibit an inherent trade-off between sensitivity and specificity (29–31). From a clinical perspective, the consistently high negative predictive value of OITS (>98%) indicates that a negative result reliably excludes clinically relevant high-grade disease. This feature supports its potential role as a rule-out test within cervical cancer screening and triage algorithms, particularly in settings where over-referral to colposcopy is a major concern or where experienced colposcopists are not readily available (32, 33).

Furthermore, the strong performance of OITS in women with negative cytology or low-grade colposcopic impressions suggests that OITS may facilitate the detection of occult high-grade lesions that would otherwise be missed by conventional methods, thereby improving patient safety without substantially increasing false-positive rates. Collectively, these findings indicate that OITS could serve as an effective adjunct or alternative triage tool following positive HPV testing, with the potential to optimize referral strategies and healthcare resource allocation. The superior diagnostic performance of OITS may be attributed to its ability to capture subtle optoelectronic and microstructural alterations associated with cervical epithelial dysplasia, which are likely to precede overt morphological changes detectable by cytology or conventional colposcopy (34–36). Unlike cytology, which relies on exfoliated cells, or HPV testing, which reflects viral presence rather than disease severity, OITS appears to provide a more direct assessment of tissue-level pathological changes (15, 16, 37). In addition, the objective and standardized nature of OITS assessment may reduce interobserver variability, a well-recognized limitation of both cytology and colposcopy. This characteristic may enhance diagnostic reproducibility and support broader implementation of OITS across diverse clinical settings.

It is important to acknowledge that the moderate specificity of OITS (~62%) may raise concerns regarding potential over-referral. However, when placed in clinical context, the specificity of OITS is substantially higher than that of hrHPV testing (4.1%) and LBC ≥ASCUS (26.6%) in the same population, both of which are widely accepted in current screening practice. The relatively high false-positive rate of OITS may lead to unnecessary colposcopic referrals in a minority of women without high-grade disease; however, the clinical consequences of such false positives are generally manageable and should be weighed against the significant benefit of OITS’s exceptional sensitivity and NPV (>98%). Clinically, OITS is best positioned as a rule-out tool: a negative OITS result reliably excludes high-grade cervical disease, while a positive result should be integrated with other clinical parameters (HPV genotype, cytology, colposcopic impression) before referral decisions are made. This approach can help minimize unnecessary procedures while ensuring that high-grade lesions are not missed.

Nevertheless, several limitations warrant consideration. First, this was a colposcopy-referred population with a relatively high prevalence of high-grade lesions. This may affect the generalizability of our results to primary screening settings, where disease prevalence is considerably lower. Therefore, caution should be exercised when extrapolating these diagnostic performance metrics to general screening populations. Second, this was a single-center study, and external validation in multicenter and diverse populations is required before broader clinical implementation can be recommended. Third, the relatively small number of invasive cancer may restrict the precision of subgroup estimates. Fourth, the cross-sectional design and relatively short study duration precluded assessment of longitudinal outcomes, such as lesion progression, regression, and post-treatment surveillance value. Future prospective studies with longer follow-up and larger, multi-center cohorts are warranted to further validate the clinical utility and optimal positioning of OITS within cervical cancer screening and triage pathways.

## Conclusion

In women referred for colposcopy, OITS demonstrated excellent and stable performance in detecting high-grade cervical lesions, characterized by high sensitivity, high negative predictive value, and favorable lesion discrimination ability. These findings suggest that OITS holds considerable promise as an adjunct triage tool, particularly for ruling out high-grade disease within the colposcopy referral pathway. However, further multicenter prospective studies are warranted to confirm these findings and better define the clinical role of OITS.

## Data Availability

The original contributions presented in the study are included in the article/supplementary material. Further inquiries can be directed to the corresponding author.
